# A combined within-host and between-hosts modelling framework for the evolution of resistance to antimalarial drugs

**DOI:** 10.1098/rsif.2016.0148

**Published:** 2016-04

**Authors:** Mathieu Legros, Sebastian Bonhoeffer

**Affiliations:** ETH Zürich, Institut für Integrative Biologie, 8092 Zürich, Switzerland

**Keywords:** drug resistance, cost of resistance, malaria, *Plasmodium falciparum*, stochastic model

## Abstract

The spread of drug resistance represents a significant challenge to many disease control efforts. The evolution of resistance is a complex process influenced by transmission dynamics between hosts as well as infection dynamics within these hosts. This study aims to investigate how these two processes combine to impact the evolution of resistance in malaria parasites. We introduce a stochastic modelling framework combining an epidemiological model of *Plasmodium* transmission and an explicit within-human infection model for two competing strains. Immunity, treatment and resistance costs are included in the within-host model. We show that the spread of resistance is generally less likely in areas of intense transmission, and therefore of increased competition between strains, an effect exacerbated when costs of resistance are higher. We also illustrate how treatment influences the spread of resistance, with a trade-off between slowing resistance and curbing disease incidence. We show that treatment coverage has a stronger impact on disease prevalence, whereas treatment efficacy primarily affects resistance spread, suggesting that coverage should constitute the primary focus of control efforts. Finally, we illustrate the importance of feedbacks between modelling scales. Overall, our results underline the importance of concomitantly modelling the evolution of resistance within and between hosts.

## 1. Introduction

Antimalarial drugs, along with vector control, are an essential pillar of malaria control throughout malaria-endemic areas [1]. In the past century, several pharmaceutical compounds have been promoted as first-line defences against *Plasmodium* in a number of large-scale control efforts. Their efficacy, however, has systematically been compromised after several years of intense usage owing to the appearance and spread of parasite strains resistant to each of these drugs [2–4].

The most recent family of drugs recommended by the World Health Organization for antimalarial chemotherapies is artemisinin and chemically related compounds [5]. Wide-scale usage of these drugs (mostly used in combination therapies) in the past decade has been associated with a sharp decline in malaria mortality and success in malaria eradication or near-eradication in several countries [6]. Concerns have, however, arisen following the observation of parasite strains in southeast Asia that present a significantly slower clearance rate when treated with several artemisinin-based treatments [7,8]. Given the current importance of artemisinin-based combination therapies in the worldwide fight against malaria, the selection and spread of these (at least partially) resistant strains would have potentially dramatic consequences for malaria control [3].

In order to be able to curb the spread of such resistant strains, the mechanisms by which these strains are selected, and transmitted in the host and vector populations in endemic areas need to be better understood. Theoretical studies of the evolution of drug resistance exist across many biological systems and for many infectious diseases, including malaria [9–12]. Given the vector-borne nature of the disease, and therefore the interactions of three organisms, parasite, vector and human host, the study of malaria transmission presents an inherently higher level of complexity compared with directly transmitted pathogens. Additionally, endemic malaria transmission is found in a variety of ecological and epidemiological settings, which consequently represent a large diversity of environments for the potential selection of resistant parasite strains.

The epidemiological dynamics of malaria, as that of many infectious diseases, has been the subject of a considerable body of theoretical work [13–15], spanning a variety of modelling scales, assumptions and techniques [16,17]. This epidemiological framework in itself provided an ideal backdrop for studies of antimalarial drug resistance, and several models have investigated the dynamics of antimalarial resistance in such a context [18–20].

Most such models can and do examine a diversity of eco-epidemiological settings, such as varying transmission intensities or treatment options. In many cases, however, the dynamics within hosts, human or mosquito, are ignored. On the other hand, some models have specifically aimed to describe the within-host dynamics of *Plasmodium* within its hosts, particularly within its human host [21–27], and its impact on resistance evolution [18,28].

In the context of this study, we argue that a separate consideration of modelling scales (between- and within-host dynamics) can impede our understanding of important aspects of malarial dynamics. In particular, the evolution of resistance to antimalarial drugs is likely to be significantly impacted by dynamics occurring at both biological levels, and by any potential interaction thereof. Under the assumption of pre-existing susceptible and resistance *Plasmodium* strains co-circulating in a given environment, selective pressures acting on resistant strains will arise at the epidemiological scale, from competition between strains for transmission among host and vector populations. At the same time, specific selective pressures will impact co-circulating strains competing at the within-host level, particularly within human hosts, as suggested by the frequent co-occurrence of multiple parasite strains within infected hosts in several (mostly endemic) areas [29,30].

It is therefore expected that disease dynamics and particularly resistance evolution will be impacted in a complex fashion by the dynamics at both levels. More generally, the notion that disease dynamics might be influenced by diverse components operating at different biological and ecological scales has recently gained recognition [31,32], including in (but not limited to) the case of mosquito-borne diseases [33]. Accordingly, there has been a growing interest in models that examine such dynamics across scales [34], and more particularly in models that disentangle the respective roles of individuals and populations in pathogen dynamics [35,36]. There is therefore substantial value in models that combine epidemiological (between-hosts) and immunological (within-hosts) scales, although concerns about the potential disadvantages of additional model complexity have been raised [37].

In that context, we present here a modelling framework that describes the epidemiology of two competing strains of *Plasmodium* transmitted within and between populations of vectors and hosts. Within this framework, we can study the dynamics of competing sensitive and resistant strains, placing the focus of the study on the spread of existing resistance (rather than the dynamics of resistance emergence). Both the within-host and the between-host scales are considered. In this particular study, the former is incorporated as a multi-strain model of parasite development and host cell infection within human hosts, and the latter as an epidemiological model of transmission between human hosts and mosquito vectors. We simulate disease dynamics at both scales simultaneously, with a particular focus on interactions between scales: how within-host infection impacts transmission events, and how epidemiological settings affect competition between parasite strains, notably co-occurrence and competition within hosts. By simulating the impacts of treatment and costs of resistance, we aim to demonstrate the importance of modelling across transmission scales and biological processes for theoretical understanding of drug resistance and, ultimately, applied strategies of resistance management and disease control.

## 2. Methods

### 2.1. Model structure overview

We present in this study a stochastic model describing the transmission and competition of two parasite strains in populations of human hosts and mosquito vectors. The overall model combines a between-hosts and a within-host component, which we present separately in §§2.2 and 2.3. In §2.4, we also describe the functions that govern the relationship between these two model components, by relating the within-host gametocyte numbers to the effective transmission rate per mosquito bite.

For better readability, each model component can be most easily described as a deterministic set of ordinary differential equations, which is what we present below for the between-hosts and for the within-host model. The final, stochastic model is then obtained by translating each deterministic component into the corresponding stochastic model, using Gillespie tau-leap algorithms. This process is detailed in §2.5, along with details of the numerical implementation of this stochastic model.

### 2.2. Between-hosts model

We follow strain-specific transmission between hosts and vectors according to a modified SI model for both human hosts and insect vectors. The dynamics of susceptible hosts (*S*_H_), infected hosts (*I*_H_), susceptible vectors (*S*_V_) and infected vectors (*I*_V_) can be described by the following equations:2.1
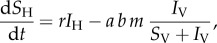
2.2
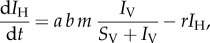
2.3
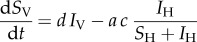
2.4
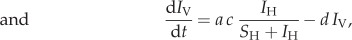
where *a* is the mosquito biting rate, *b* is the transmission probability per infectious bite from vector to host, *c* is the transmission probability per infectious bite from host to vector, *d* is the death rate for mosquitoes, *r* is the recovery rate of infectious hosts and *m* is the ratio of mosquitoes to hosts in the simulated population:2.5
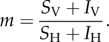


We limit this study to short epidemiological time scales and therefore ignore human demographics and age structure. We also consider the vector population size to remain constant, with a birth rate equal to the death rate *d*. This death rate applies equally to susceptible and infected vectors (the effective birth rate is therefore equal to *d*(*S*_V_ + *I*_V_), partly simplified in equation (2.3) with the death rate –*dS*_V_). All parameters and their default values are listed in table 1. Effective transmission rates *b* and *c* are determined by intrahost dynamics, as described in §2.4.

**Table 1.** List of parameters and default values. Parameters are separately listed for between-hosts and within-host models. Listed default value is the value used in this study unless otherwise specified. Parameters with subscript *i* are strain-specific (strain *i* with *i* ∈ {1, 2}, 1 is sensitive, 2 is resistant). When only one value is provided for strain-specific parameters, the same value was used in this study for both strains. Note, however, that model structure allows for strain-specific distinct values in future work.

parameter	description	default	reference
between-hosts model			
*a*	vector biting rate	0.25 d^−1^	[13], [14]^a^, [15], [38]
*b*	transmission rate from vector to host	0.3 d^−1^	
*c*	transmission rate from host to vector	0.3 d^−1^	
*d*	vector death rate	0.12 d^−1^	
*r*	host recovery rate	0.01 d^−1^	
*T*	extrinsic incubation period	14 d	
within-host model			
*Λ*	rate of uninfected erythrocyte production	1	[22]^a^
*μ_x_*	death rate of uninfected erythrocytes	1/120 d^−1^	[22]^a^, [39]
*μ_y_*	death rate of infected erythrocytes	0.5 d^−1^	[22]^a^, [39]
*μ*_s_	death rate of free merozoites	1/20 min^−1^	[22]^a^, [39]
*μ_g_*	death rate of circulating gametocytes	1/16 d^−1^	[22]^a^, [40]
*μ*_I_	death rate of immune cells	1/20 d^−1^	[22]^a^
*β_i_*	infection rate of erythrocytes by free merozoites	0.1	[22]^a^
*η_i_*	gametocyte formation rate	0.02	[22]^a^
*ρ_i_*	number of merozoites produced per infected erythrocyte	16	[14]^a^, [22]^a^
*k_i_*	efficacy of immune response against infected erythrocytes	0.05	[22]^a^
*h_i_*	efficacy of immune response against free merozoites	0.05	[22]^a^
*l_i_*	efficacy of immune response against gametocytes	0.05	[22]^a^
*γ_i_*	immune stimulation by infected erythrocytes	0.1	[22]^a^
*σ_i_*	immune stimulation by free merozoites	0.1	[22]^a^
*λ_i_*	immune stimulation by gametocytes	0.1	[22]^a^
*Θ*	background rate of immune cells production	0.01	[22]^a^
*ϕ_i_*	strain-specific cost of resistance (within-host reduction of merozoites production)	*ϕ*_1_ = 0; *ϕ*_2_ = 0.01	
*ɛ_i_*	strain-specific treatment efficacy (within-host increase of infected erythrocytes mortality)	*ɛ*_1_ = 0.9; *ɛ*_2_ = 0	
*Σ*	treatment coverage (among hosts)	0.9	

^a^And references therein (refers to key modelling studies; where empirical support for parameter values was lacking, default values in this study were chosen to match those used in these specific articles).

We implement stochastic simulations of host and vector population dynamics based on this model in a fashion described in §2.5. In these simulations, infection status is tracked for each host and vector individually. For each transmission event, the date and involved parasite strain are recorded, and within the affected hosts, the parasite dynamics are modelled as described in §2.3.

### 2.3. Within-host model

The dynamics of multi-strain infection within the human host can be described by the following set of equations (equations (2.6)–(2.10)), adapted from earlier theoretical work [22,24]:2.6
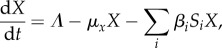
2.7
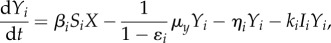
2.8
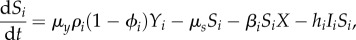
2.9
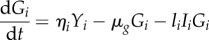
2.10
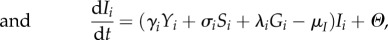
where *X* represents uninfected erythrocytes in the host and constitutes the resource for which (up to two) parasite strains compete. *Y_i_*, *S_i_* and *G_i_* represent infected erythrocytes, free merozoites and circulating gametocytes, respectively, for parasite strain *i* (*i*∈{1, 2}). Finally, *I_i_* represents the strain-specific immune component against strain *i*. The inclusion of this strain-specific immunity allows co-infection and co-circulation of parasite strains with different fitnesses [22]. This immune component is not meant to represent any particular mechanism of human immunity, but represents the action of the host immune system as a whole on circulating parasites of a specific strain.

Uninfected erythrocytes *X* are produced at a constant rate *Λ*, and die at a rate *μ_X_*. Erythrocytes are infected by free merozoites *S_i_* of strain *i* at a rate *β_i_S_i_* to produce infected cells *Y_i_*. These infected cells die at a rate *μ_y_* producing in the process *ρ_i_* free merozoites. Infected erythrocytes can also produce gametocytes at a rate *η_i_*. Free merozoites and gametocytes die at a rate *μ_s_* and *μ_g_*, respectively. All parasite stages are affected by an immune component *I_i_* acting against the corresponding strain. Infected erythrocytes, free merozoites and gametocytes die at rates *k_i_I_i_*, *h_i_I_i_* and *l_i_I_i_,* respectively. Immune compartment *I_i_* is in turn boosted by this reaction, at respective rates *γ_i_Y_i_*,*σ_i_S_i_* and *λ_i_G_i_*. Immune cells die at a constant rate *μ*_I_ and are produced at a constant rate *Θ*. Treatment is assumed to act on parasite replication inside erythrocytes. We therefore model the impact of treatment as an increase in the mortality rate of infected erythrocytes by a factor (1−*ɛ_i_*)^−1^, where *ɛ_i_* corresponds therefore to the strain-specific treatment efficacy. Treatment is modelled as a binary attribute of an infected host. The probability that an infected host receives treatment is defined by treatment coverage *Σ*, and the treatment status of a given host remains until the host is removed from the infected compartment (*I*_H_).

Finally, we define strain-specific costs *ϕ_i_*, in particular for addressing costs of resistance. While the precise stage at which such costs may appear is not well defined, there is evidence that the growth of the parasite at the erythrocytic stage is affected [41,42]. We therefore elect to apply these costs to the number *ρ_i_* of free merozoites produced per infected erythrocyte, which is therefore scaled by a factor (1−*ϕ_i_*).

Within-host dynamics of the parasite in the final model are stochastically simulated based on these equations, as described in §2.5. Note that, for simplicity, we do not model the intrahost dynamics within the mosquito vector explicitly. We consider, instead, a fixed duration *T* as the incubation period within the mosquito (known as the extrinsic incubation period). An infected mosquito is considered infectious (i.e. can potentially infect susceptible human hosts) only after this incubation period *T* has elapsed after that mosquito became infected.

All parameters and their default values are listed in table 1.

### 2.4. Interface between model components

As the course of infection with multiple strains is followed within each host, the values of within-host state variables at the time of contact between host and vector can influence the outcome of this contact, and therefore impact the occurrence of transmission events at the between-host scale.

In the case of contact between an infected vector and a host, the time lapsed since the vector was first infected is checked. If this time exceeds the extrinsic incubation period *T*, infection of the host may occur. The rate of infection is then *c* as defined by the between-host model.

**Figure 1.**
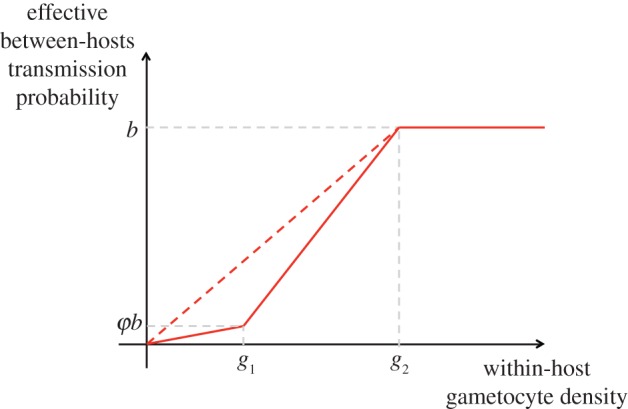
Gametocyte-dependent transmission probability in a contact between vector and infected host. The effective transmission probability is defined by equation (2.11) in the main text, and corresponds to the solid line in this figure. Two gametocyte-dependent thresholds are defined, *g_1_* and *g*_2_. For gametocyte concentration beyond *g*_2_ the probability remains at the value defined in the between-hosts model, *b*. The dashed line corresponds to the case where *φ* = *g*_1_/*g*_2_. In this case, there is effectively no lower threshold to gametocyte-dependent transmission probability. (Online version in colour.)

In case of contact between a vector and an infected host, the number of circulating gametocytes *G* in the host is checked. There are few studies that explicitly investigate the relationship between concentration of circulating gametocytes and infectivity to susceptible vectors. Consequently, the precise form of this relationship is not well defined. In this study, we have followed the observations of the most recent and most detailed study on this topic [43] to define the realized transmission rate b* as follows (see also figure 1):2.11
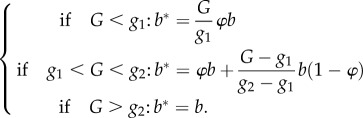

With a low default value of *φ* = 0.1, this corresponds to two thresholds for transmission: one low threshold *g*_1_ under which transmission rate remains low (increasing linearly from 0 to *φb*), one high threshold *g*_2_ above which transmission rate is maximal at *b* (given by the between-host model) and is not sensitive to the gametocyte concentration (figure 1). Between the two thresholds transmission rate increases linearly at a steeper slope, reproducing the pattern observed in empirical studies [43].

### 2.5. Numerical implementation

We run stochastic simulations of the full model, including between-hosts and within-host components of the model. Fixed numbers of hosts and vectors are individually followed throughout the simulation (each population retains a constant size, because human demography is ignored and vector deaths are compensated by births of susceptible vectors). For each model, the corresponding differential equations are stochastically simulated following Gillespie's algorithm with tau-leaping method [44]. In this framework, multiple similar events (defined in the respective model descriptions) can occur within the same time step; in that case the number is drawn from a Poisson distribution with a mean given by the product of the event defined rate and the actual time step.

The model is implemented in C++. Unless otherwise noted, simulations use a time-step of 1 h at the between-host level, of 1/12 h at the within-host level, and consider a population of 10 000 hosts (the vector population is sized according to the value of *m*). The resistant strain is introduced after a burn-in running period of 1 year, at an initial frequency in the host population of 1%, and the model is then run for a time span of 5 years. At this resolution and for this stochastic model, our ability to run replications is inevitably limited by computational constraints. We typically choose to run 30 identical simulations (with same parameter values and same initial conditions) for a given parameter set.

## 3. Results

We first focus on the interactions between scales in our model, and how competition between strains within human hosts can be influenced by epidemiological dynamics. Different epidemiological settings are primarily characterized by different entomological inoculation rates (EIR, the most widely used metric of transmission intensity, defined as the expected number of infected bites received by a host in a given period of time). In our model, we can directly vary this EIR by changing the value of *m*, the vector-to-host ratio, which is directly proportional to EIR. We can therefore also interpret results of varying *m* as scenarios with varying EIR.

**Figure 2.**
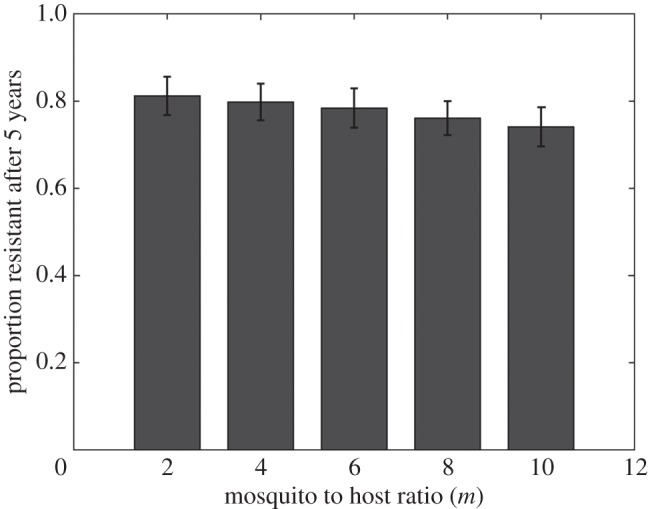
Resistance spread and mosquito to host ratio. Proportion of resistant parasites after 5 years in simulations with resistant parasites introduced into a sensitive population. Parameters are set at default value as defined in table 1. Number of hosts: 10 000. Number of vectors: defined by *m* the mosquito to host ratio. Average of 30 simulations (±s.e.).

We find that in high transmission settings, the proportion of resistant parasites in the simulated population after 5 years is lower (*F* = 13.12, *p* < 0.001, logit-transformed data) than in low transmission settings (figure 2), although the effect remains relatively weak with the default value of the cost of resistance (*ϕ*_2_ = 0.01). Interestingly, vector control approaches, which constitute a major component of the fight against malaria, aim primarily to reduce the value of this ratio *m*. This result would imply that such measures, while efficient at curbing disease incidence, could have the undesired side effect of speeding up the spread of pre-existing resistant parasite strains.

**Figure 3.**
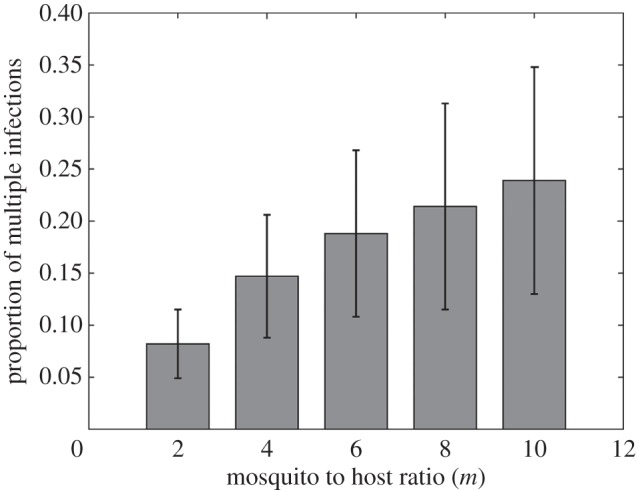
Multiple infections and mosquito to host ratio. Proportion of multiple infections in human hosts through 5 years in simulations with resistant parasites introduced in a sensitive population. Parameters as in figure 2. Average of 30 simulations (±s.e.).

One potential underlying mechanism for this slower spread of resistance in high transmission areas is the impact of competition between strains within a host, based on the assumption that a resistant strain would be less fit than a sensitive strain in the absence of treatment. We illustrate this impact by confirming that multiple infections (co-circulation of two distinct strains in a given host) are significantly more frequent (*F* = 17.47, *p* < 0.001, logit-transformed data) when transmission intensity is higher (figure 3).

**Figure 4.**
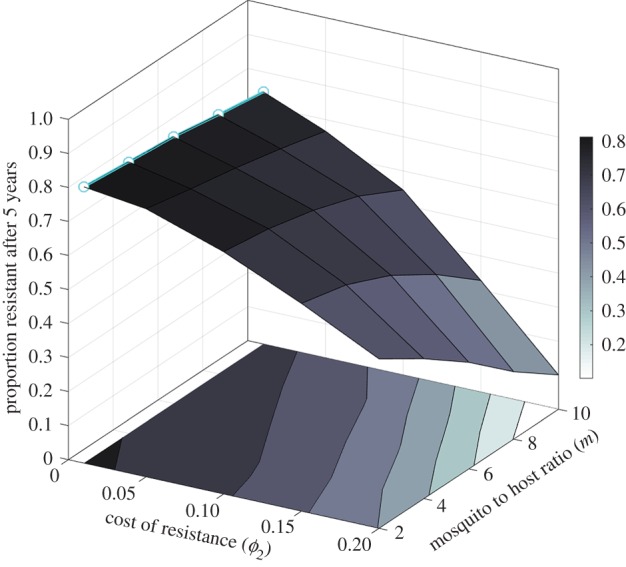
Interactions between cost of resistance and mosquito to host ratio. Proportion of resistant parasites after 5 years in simulations with resistant parasites introduced into a sensitive population. Parameters as in figure 2 except cost of resistance *ϕ*_2_ defined for the resistant strains (no cost *ϕ*_1_ = 0 for the sensitive strain). Average of 30 simulations, s.e. not shown. Surface shading reflects levels on the *z*-axis (see values on the colour bar along the *z*-axis). The cyan line (with circle symbols) marks the data from figure 2. Contour plot is drawn on the graph floor. (Online version in colour.)

If the above explanation is correct, the significance of this effect should increase with the difference in fitness between both strains, that is, with the cost of resistance. We show that this is indeed the case, and that higher costs of resistance not only impede the spread of resistance as expected, but also exacerbate the impact of a higher mosquito-to-host ratio and the difference between high and low transmission settings (figure 4, variance levels not pictured but quantitatively comparable to those in figure 3).

**Figure 5.**
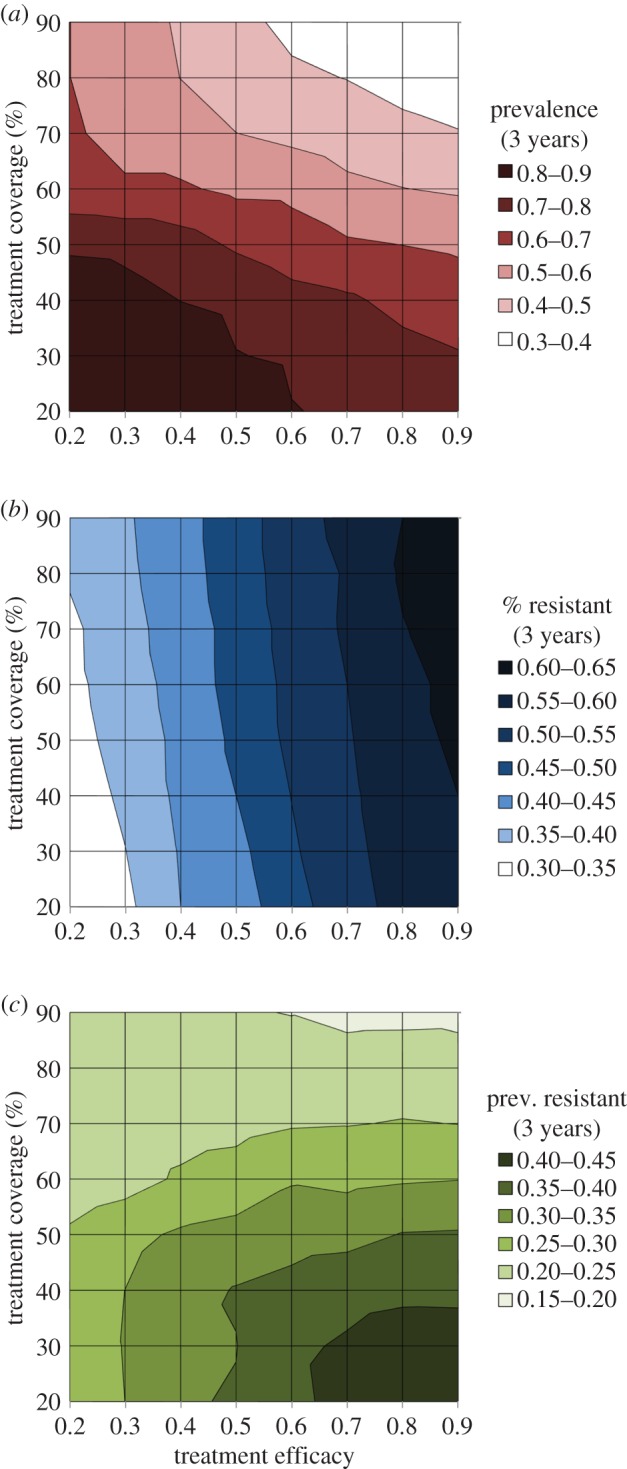
Impact of treatment on disease prevalence and spread of resistance. Effect of treatment efficacy and treatment coverage (as defined in the text) after 3 years in simulations with resistant parasites introduced into a sensitive population on (*a*) disease prevalence (given by surface shading), (*b*) proportion of resistant parasites, and (*c*) prevalence of resistant parasites (product of panels *a* and *b*). Other parameters set at default values given in table 1. Average of 30 simulations, s.e. not shown. (Online version in colour.)

We also investigate the impact of treatment on the dynamics of infection in our simulated populations. As described in §2, we consider treatment to vary in both its efficacy (within-host) and its level of coverage (between-hosts). We show that disease prevalence is lower when efficacy and coverage are increased (figure 5*a*), and that resistance levels (sampled after 3 years to better emphasize differences that occur during the rise of the resistant strain in the population) are higher under the same conditions (figure 5*b*). This implies again a trade-off between disease control and resistance, where a decrease in disease incidence appears to be systematically associated with higher levels of resistance among the remaining parasites. Interestingly, the results show that disease prevalence is most sensitive to treatment coverage, whereas the levels of resistance are markedly more sensitive to treatment efficacy. This suggests that at high levels of treatment coverage, the impact on disease prevalence would be stronger with only a weak impact on resistance levels (figure 5*c*), mitigating the undesirable impact of the above-mentioned trade-off.

**Figure 6.**
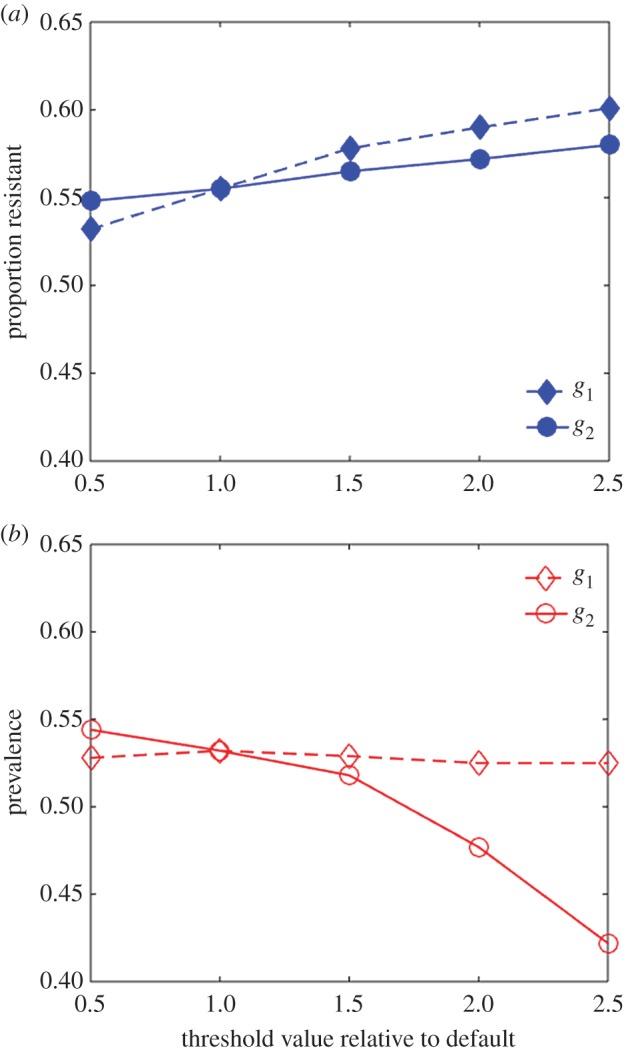
Sensitivity of proportion of resistance and disease prevalence to gametocyte-dependent transmission thresholds. Proportion of resistant parasites (*a*) and prevalence among hosts (*b*) in simulations with resistant parasites introduced into a sensitive population. Dashed lines: parameter *g*_1_ is varied, the low density transmission threshold as described in equation (2.11) (see also figure 1). Solid lines: parameter *g*_2_ is varied, the high density threshold. Threshold values are plotted relative to default (i.e. 1 is default value). Average of 30 simulations (s.e. not shown.) (Online version in colour.)

A key component of this multi-scale model is the quantitative interface between these scales, that is, the calculation of an effective transmission rate between an infectious host and a naive vector based on the gametocyte concentration circulating in the human host (equation (2.11)). We define two thresholds in gametocyte concentration, and both disease prevalence and proportion of resistant parasites are sensitive to these two thresholds (figure 6). More precisely, the proportion of resistant parasites increases when either threshold is raised, whereas disease prevalence decreases when the high transmission threshold *g*_2_ is raised. Interestingly, disease prevalence appears to be sensitive only to *g*_2_ and not to the low transmission threshold *g*_1_, whereas the fraction of resistant parasites is shown to be more strongly impacted by the lower threshold *g*_1_.

**Figure 7.**
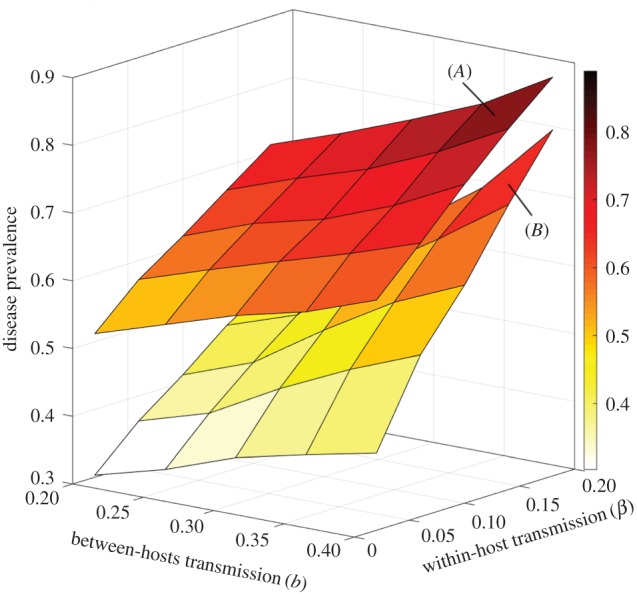
Sensitivity to within-host and between-hosts transmission parameters. Disease prevalence after 5 years in simulations with resistant parasites introduced into a sensitive population, as a function of between-hosts host to vector transmission parameter (*b*) and within-host erythrocyte infection parameter (*β*). Bottom surface (*B*): default values of *φ*, *g*_1_ and *g*_2_. Top surface (*A*): *φ* = *g*_1_/*g*_2_. In this latter case, there is no lower threshold to gametocyte-dependent host to vector transmission. Surface shading reflects levels on the *z*-axis (see values on the colour bar along the *z*-axis). Average of 30 simulations (s.e. not shown). (Online version in colour.)

Overall, we show with this model that disease dynamics depend in a complex fashion on transmission patterns between hosts as well as parasite infection dynamics within hosts. Classic disease dynamics parameters can therefore not apply to this model, but need to be considered at each separate scale. For example, parasite transmission is chiefly governed by two parameters: the within-host ability of the parasite to replicate and infect new erythrocytes (*β_i_*), as well as the between-hosts ability to transmit from an infected to a susceptible human host (*b*). Disease prevalence in the model is positively correlated with both parameters (figure 7), but there is a clear interaction between these scales: the impact of within-host replicative ability is much stronger at high between-host transmission probabilities. It should be noted that this interaction appears to be dependent on our specific, nonlinear interaction between gametocyte density and effective transmission rate. When this nonlinearity is partly removed by setting *φ* = *g*_1_/*g*_2_ (illustrated in figure 1), the interaction between *b* and *β_i_* appears less pronounced (figure 7, variance levels not pictured, but quantitatively comparable to previous scenarios, see figure 3 for example).

## 4. Discussion

The evolution of drug resistance has been studied in many disease systems, including *P. falciparum* malaria. We introduced in this study a model combining within-host and between-hosts modelling scales, and we have shown that the dynamics of two competing strains, sensitive and resistant, depend on specific assumptions at each of these scales and on interactions between dynamics across scales. While previous studies have investigated the epidemiological aspects of resistance, and some studies have focused on the within-host dynamics of resistance, the interactions between these biological processes remain incompletely characterized. The modelling framework presented in this study, studying malaria dynamics across biological scales, provides important insights into treatment practices and disease control, and more generally demonstrates the value of examining disease dynamics and the evolution of resistance with consideration for between- and within-hosts modelling scales.

We have shown that the competition between sensitive and resistant strains, which occurs primarily within the human host, directly impacts the spread of resistance at the population level. Interestingly, there is a clear interaction between traits of the resistant strain at the within-host level (cost of resistance) and population-level epidemiological traits (ratio of vectors to host) in determining the fate of a resistant strain in a population. In particular, we show that resistance does not spread as fast in the population in areas of higher transmission intensity. Other studies have described an effect in the opposite direction [18,28], and it is likely that details about assumptions on competition as well as treatment explain the observed discrepancies. We point out in particular (i) that by explicitly modelling the within-host dynamics of different life stages of the parasite, we place a stronger emphasis on the outcome of competition for erythrocytes, which is more relevant during the acute phase of the infection but less so during the transmission phase and (ii) that by representing treatment as a binary attribute of an infected individual we ignore potentially important effects of within-host pharmacodynamics and pharmacokinetics, and associated strain-specific impacts.

Nevertheless, these results clearly illustrate the importance of considering a combination of scales in a theoretical study like this one, and underline the level of complexity involved in predicting the evolution of resistance in malarial parasites. For instance, the ratio of vector to hosts is known to vary greatly across malaria-endemic areas, while the cost of resistance remains very poorly quantified, particularly for resistance to artemisinin-based therapies. The sensitivity illustrated in this study demonstrates, therefore, the importance of gaining better quantitative understanding of both these aspects of malaria transmission and infection.

This interaction between scales also provides crucial insights into the impact of treatment on disease dynamics and resistance evolution. We have shown that treatment efficacy, considered primarily as the ability to kill parasites in the blood or in blood cells, and therefore a within-host parameter, and treatment coverage, considered as the probability for a host to receive treatment, and as such a parameter of the between-host level, have impacts that interact in a complex fashion. Interestingly, while the potential negative impacts of strong disease control on resistance levels have been previously examined [45], with this multi-level approach we show that this trade-off operates more or less strongly for different types of treatment. Treatment coverage and efficacy appear to be at odds, analogously to previously described effects for other types of intervention, particularly vaccines [46,47]. In our results, coverage seems to be the more crucial aspect, as it strongly impacts disease prevalence while only moderately favouring the spread of resistance, even at high coverage levels. This would suggest that, in a hypothetical situation where improving treatment coverage and efficacy at the same time would not be feasible, a focus on high treatment coverage would most benefit disease control, and mitigate to some extent the undesirable effects of the aforementioned trade-off, namely the selection for resistant strains.

More generally, we observe that the results of this multi-scale model are substantially impacted by both modelling levels, and appear sensitive to specific assumptions and parameter values at each scale. These results, while preliminary, seem to suggest that none of these biological processes can easily be ignored in modelling studies, particularly when examining issues of drug resistance evolution. Furthermore, the results emphasize the importance of processes occurring across scales (e.g. here, competition), and of specific assumptions regarding the interface between these scales. This highlights not only the importance of the methodological fringe zone between within- and between-hosts modelling approaches, but also emphasizes the crucial epidemiological links between individual infectiousness and transmission intensity. The results of our model are highly sensitive to assumptions regarding this specific juncture (figure 7) in the same way that our overall understanding of malaria epidemiology and control hangs on those biological relationships [48].

Methodologically, it should be pointed out that the model presented here is a fairly complex model with a high number of parameters. While this approach was chosen for its mechanistic merits in describing the dynamics of *Plasmodium* parasites (particularly within its human host), high dimensionality poses significant challenges that can potentially offset these benefits, a classical trade-off for epidemiological and ecological modelling [37,49].

The most significant challenges concern model parametrization. When empirical studies have provided estimates for any given parameter in this model, we have based our parametrization choices on these studies. Some often-studied parameters can therefore be set with reasonable levels of confidence. This includes most parameters of the between-hosts model (although often highly variable between transmission areas), and within-host parameters such as the gametocyte production rate *η*_*i*_, their mortality rate *μ_g_* (the lifespan of gametocytes has been empirically measured by several studies), and the number of merozoites produced by an infected erythrocyte. Other parameters of within-host model, while not directly measurable, can be inferred from empirically measurable proxies. This includes, for example, the erythrocyte infection rate *β*, and the lifespan of infected erythrocytes (*μ_y_*^−1^).

Yet many parameters of the within-host model are at present not supported by any empirical study, and can therefore only be parametrized ad hoc in a fashion that supports reasonable infection dynamics within a given host. This is primarily the case of parameters pertaining to immune action against specific parasite stages (*h_i_*, *k_i_*, *l_i_*, *γ_i_*, *σ_i_* and *λ_i_*,). While the competition between parasite strains in our framework is primarily driven by the availability of uninfected erythrocytes, the immune component was included in the model to allow for the frequency-dependent competition and therefore co-circulation of competing strains. As such, it is limited to a simplistic impact on parasite dynamics, and cannot be considered to provide a faithful representation of the complex interactions existing between host immunity and *Plasmodium* dynamics (within and between hosts). The current modelling framework could however be further extended to include a more detailed description of host immunity, particularly with regards to immunity acquisition through successive infections. This extended model would further elucidate the potential impacts of immunity on strain-specific transmission and ultimately on the selection for resistance.

More generally, even in its current form our model relies on a number of parameters, and the sensitivity of the results to each parameter and model component should be further elucidated. In this study, we have focused on sensitivity to eco-epidemiological parameters (i.e. between-host model parameters), as well as to those specific elements that define the interaction between model components. With these results, we emphasize the importance of considering disease dynamics across biological scales, which constitutes the central point of this article. To better characterize the impact of within-host dynamics in particular, further in-depth sensitivity analyses will be valuable, and are part of our future plans with this modelling framework. The value of such analyses is further emphasized by (i) the natural level of variation in several aspects of the model, whether epidemiological [15,38] or immunological [24] and (ii) the inherent level of uncertainty in many aspects of the model, particularly in parameters describing within-host dynamics and the host immune response. For these reasons, large-scale sensitivity and uncertainty analyses, although methodologically complex with high-dimensional models such as this one, will provide further insights into the respective roles of within- and between-hosts processes (and their potential interactions) in disease dynamics and resistance evolution.

At this stage, our modelling framework presents significant limitations, and is therefore not able to capture and describe the variety of epidemiological dynamics that *Plasmodium falciparum* exhibits in natural populations. In this article, we deliberately chose simplistic scenarios that allow us to examine the impact of a few specific parameters, but at the cost of realism when comparing with specific field situations. In particular, we consider short epidemics where resistance is initially present at relatively high levels, is completely impervious to treatment and rises therefore in a short period of time (only a few years). Another significant limitation of the current framework is the restriction to two circulating strains, sensitive or resistant. We ignore at this stage more complicated genetic architectures, notably the inclusion of multiple resistance loci and the impact of recombination, which has been shown to have a significant impact on the evolution of multiple drug resistance [50,51]. We also cannot elucidate in this framework the specific dynamics of resistance emergence.

In further studies, we aim to extend our modelling framework to consider more elaborate multi-loci and multi-drug dynamics. Our current framework across scales will be particularly appropriate to study these questions, because mixed infections within hosts represent a crucial part of these dynamics. Generally, the rationale for the high-dimensional, stage-specific parametrization of this model is to set the stage for more complex and/or realistic studies of malaria transmission and control (including vector control). In this framework, however, a better quantitative knowledge of several key aspects of *Plasmodium* dynamics, most notably of the host immune action against various stages of the parasite, is required.

Informed by such empirical data across biological scales, we believe that the combined modelling approach presented in this study will provide an improved framework to further our understanding of disease dynamics with competing mixed infections, and the resulting consequences for the evolution of drug resistance.
